# Artificial intelligence: a useful tool in active tuberculosis screening among vulnerable groups in Romania - advantages and limitations

**DOI:** 10.3389/fpubh.2025.1433450

**Published:** 2025-02-07

**Authors:** Beatrice Mahler, Alexandru Stoichiță, Dragoș Băiceanu, Traian-Constantin Panciu, Dragoș Dendrino, Mihaela Mihai, Raluca Bobocea, Elmira Ibraim, Mara Bălteanu, Oana Popescu, Mădălina Oana Burecu, Ioana Mădălina Moșteanu, Vanessa Veronese, Radu Matache, Ioana Munteanu, Cristina Popa, Justina Antonela Dragomir

**Affiliations:** ^1^Pneumology II Department, Faculty of Medicine, "Carol Davila" University of Medicine and Pharmacy, Bucharest, Romania; ^2^Pneumology Clinic, "Marius Nasta" Institute of Pneumology, Bucharest, Romania; ^3^Tuberculosis Screening Program, "Marius Nasta" Institute of Pneumology, Bucharest, Romania; ^4^Research Department, "Marius Nasta" Institute of Pneumology, Bucharest, Romania; ^5^Faculty of Management, Bucharest University of Economic Studies, Bucharest, Romania; ^6^Department of Statistics and Econometrics, Faculty of Economic Cybernetics, Statistics and Informatics, The Bucharest University of Economic Studies, Bucharest, Romania; ^7^Faculty of Medicine, "Titu Maiorescu" University, Bucharest, Romania; ^8^Faculty of Medicine, University of Medicine and Pharmacy of Craiova, Craiova, Romania; ^9^The Special Programme for Research & Training in Tropical Diseases, World Health Organization, Geneva, Switzerland

**Keywords:** tuberculosis, artificial intelligence, computer-aided detection, Romania, screening, vulnerable populations

## Abstract

**Introduction:**

Despite advances in diagnostic technologies for tuberculosis (TB), global control of this disease requires improved technologies for active case finding in selected vulnerable populations. The integration of artificial intelligence (AI) into imaging modalities has been anticipated to assume a pivotal position in conjunction with traditional bacteriological diagnostic approaches, especially in the active diagnosis of vulnerable groups.

**Methods:**

The study was conducted as a prospective investigation spanning from November 2019 to October 2023, in Romania’s national TB screening project. From total of 92,368 tested participants, 404 patients were included in the study, with 202 individuals diagnosed with TB and 202 individuals serving as controls. The initial interpretation of radiological images was performed by AI X-Vision software and patients with suspicious findings were confirmed to have TB using GeneXpert testing. The objective of this study is to discover a threshold at which the AI score can accurately assess the risk of TB, regardless of the patient’s medical background.

**Results:**

The study involved a number of 404 people, among whom 202 were diagnosed with TB out of a total of 92,368 participants, and the remaining 202 patients represent the control group. The current study highlighted, at an AI threshold value of 0.4, that 89% of the screened people benefited from a correct assessment using the AI associated with the radiological examination. ROC curve analysis indicates an AUC of 0.923 (95% CI:0.893–0.947; significance level *p* < 0.0001) which shows that the test has a high capacity to accurately detect individuals with TB and also to rule out those who do not have the disease, with sensitivity 87.1, specificity 91.6 and criterion >0.3585.

**Conclusion:**

Our study brings to the fore the significance of integrating AI software X-vision with bacteriological diagnosis in detecting TB among vulnerable groups in Romania. This underscores the imperative at the global level to develop solutions in the prompt diagnosis of TB, particularly within vulnerable groups.

## 1 Introduction

Approximately one-quarter of the global population is estimated to harbor *Mycobacterium tuberculosis*, the bacterium responsible for TB disease. In 2022, there were 10.6 million reported cases of TB, with 7.5 million being newly diagnosed, suggesting that approximately 3 million cases were missed by health systems. Despite being both preventable and treatable, TB resulted in around 1.3 million deaths that year, with the burden falling disproportionately on marginalized populations—over 80% of cases and fatalities occur in low-and middle-income countries. Currently, we are not on track to meet the Sustainable Development Goal 3 (SDG 3), which aims to eliminate TB as a public health threat by 2030. Historically, TB has struggled due to inadequate funding and attention, even as it remains one of the deadliest infectious diseases worldwide (1).

There is a notable disparity between the estimated figures and the officially reported numbers, and this divergence has been exacerbated amidst the COVID-19 pandemic. The early identification of subjects with active TB, expeditious identification of drug resistance, and initiation of an effective treatment regimen are all imperative for the appropriate management of TB (2). Active case finding by screening of vulnerable populations plays a crucial role in the management of TB, serving as a fundamental component of the first pillar of the End TB Strategy. This pillar strives to ensure the early identification of all individuals affected by TB. The primary goal of the United Nations and the World Health Organization (WHO) is in line with the objectives of the END TB strategy, which seeks to decrease the occurrence of TB to below 20 cases per 100,000 individuals by the year 2030 (3, 4).

Romania is classified as one of the 18 high-priority nations for TB control within the European Region by the WHO. In 2020, the incidence rate of TB in the country was the highest among all European Union (EU) member states, with a rate of 64 cases per 100,000 individuals. This accounted for 23% of all TB patients within the EU. It is noteworthy that despite a declining trend in TB incidence, with an average annual reduction rate of 5.5%, the country continues to experience a significant burden of the disease (5–9).

Given the advancements in technology, the incorporation of AI into imaging techniques is expected to play a crucial role in identifying lung lesions associated with infectious diseases such as TB and severe acute respiratory syndrome coronavirus (SARS-CoV-2) alongside conventional diagnostic methods (10).

The recently published WHO consolidated guidelines on TB screening in 2021 have introduced a novel recommendation. Specifically, it suggests the utilization of computer-aided detection (CAD) software as a substitute for human readers in the interpretation of digital chest radiography (CXR) for individuals aged 15 years and above. This recommendation applies to both TB disease screening and triage purposes. AI algorithms produce a continuous abnormality score (from 0 to 100 or from 0 to 1) that represents the likelihood of the presence of TB-associated abnormalities (11).

The utilization of CXR is of paramount importance in the process of screening and triaging individuals suspected of having pulmonary TB. It serves as a valuable tool in directing the appropriate utilization of diagnostic testing, thereby enhancing the identification of TB cases and optimizing cost-effectiveness. The utilization of CAD solutions has the potential to enhance the feasibility and efficiency of CXR in the context of TB screening and triage. This is achieved through the application of AI algorithms that analyze CXR images to identify abnormalities that may indicate the presence of pulmonary TB. In comparison to a selected threshold, CAD products produce an anomaly score that can be utilized to signify the potential presence of TB and prompt additional diagnostic examinations (12).

Romania adheres to the guidelines set forth by the WHO for the timely identification and diagnosis of TB. This commitment is demonstrated via the implementation of several initiatives and the subsequent adoption of the National Strategy for Tuberculosis Control, which includes provisions for TB screening (13). Romania has adopted CAD technology for TB screening since 2018, which was performed on a smaller number of patients, and for a shorter duration (12).

The utilization of CAD yields numerous advantages in the screening process, with the selection of the TB risk threshold being pivotal in distinguishing TB patients or the potential risk of missing them during screening. The significance of the population being addressed and their accessibility to this medical service is crucial in this regard. While evaluating a project’s direct financial costs is essential, it is equally important to recognize the indirect benefits that may arise from the initiative. Interventions targeting vulnerable groups are crucial for eliminating TB in Europe, as stated by the European Centre for Disease Prevention and Control (14, 15). The threshold scores exhibit considerable variation across different CAD software packages, and this variability can also be observed between newer iterations of the same software program.

This research aims to evaluate the sensitivity and specificity of the X-Vision software, as well as the risk of false-positive or false-negative results, among vulnerable groups diagnosed with TB, such as the homeless, people struggling with alcohol addiction, drug users, prisoners and those residing in rural areas.

The main objective of this study is to analyze the results of using the X-vision software in patients from vulnerable groups to establish the method’s risk threshold regarding the loss of active TB cases during screening, as well as the risk of over diagnosis based on the use of a workflow targeting CAD technology and GeneXpert testing. The effectiveness of CAD is contingent upon both the selected threshold limit and the software’s capacity for refinement during testing, using mathematical algorithm methods.

## 2 Materials and methods

### 2.1 Study design

The study design encompasses a retrospective case–control study which integrates data acquired from the TB screening program conducted in Romania between November 2019 and October 2023.

### 2.2 Study population

#### 2.2.1 Inclusion criteria

The study population comprises individuals aged 18 years and above who underwent a TB screening by the National Screening Project, carried out between November 2019 and March 2023. The screened population consists of individuals from four vulnerable populations: the homeless (HP), people who inject drugs (PWID), rural residents (RR), persons deprived of liberty (PDL).

A case of TB was defined as a person who had a CAD score greater than 0.40, associated with a confirmation of the disease by the GeneXpert test. People with a score lower than 0.40 and negative GeneXpert tests were defined as cases belonging to the control group, being chosen randomly by the computer software used.

#### 2.2.2 Exclusion criteria

Pregnant women or participants with medical conditions that are not compatible with performing a CXR and children under 18 years old. Individuals who were non-speakers of the Romanian language and participants who declined to partake will be excluded from the study.

### 2.3 Sampling and recruitment

This study evaluates two patient cohorts, each comprising 202 individuals, namely a study cohort and a control cohort. The study group comprises patients who were diagnosed with TB based on a radiological AI score greater than 0.40 and confirmed by bacteriological tests using GeneXpert. The control group consists of patients randomly selected by computer from a total of 92,166 screened subjects.

The recruitment process for the study population was designed to ensure representation from the four identified vulnerable groups defined in the inclusion criteria: HP, PWID, RR and PDL. Participants were recruited through partnerships with local healthcare providers, social services, non-governmental organizations, and community outreach programs. Screening caravans visited rural areas and homeless shelters, while correctional facilities enabled access to incarcerated individuals. For people who inject drugs, collaborations with rehabilitation centers ensured participation.

To minimize selection bias, random sampling was employed within each group using computer-based algorithms. For the RR, screening targeted areas with the highest number of TB cases recorded in the 5 years preceding the study, aligning with local public health priorities. For HP, PWID and PDL, all individuals identified within the screening period were invited to participate.

Practically, the diagnostic caravans performed CXR, and the score greater than 0.40 was followed by sputum collection for GeneXpert testing. These patients were subsequently followed up by the National Tuberculosis Surveillance and Control Network.

Patients with a score below 0.40 were not tested bacteriologically, unless further reading of the radiograph raised a physician’s suspicion of TB and required further investigations.

Following national screening, 216 patients were diagnosed with TB using only CXR with CAD and GeneXpert testing. Data analysis will focus in this study on the concordance between positive results obtained from CAD and the reference standard of GeneXpert testing in identifying actual TB cases. Also, CAD cases showing positive results in sputum culture for *Mycobacterium tuberculosis* complex (MTB) will be classified in this category even if their GeneXpert results are negative.

Another aspect to be analyzed is the occurrence of false positive cases, especially when there is a disparity between the positive results from CAD testing and the negative results from GeneXpert testing. Cases are considered true negatives when both CAD and GeneXpert tests yield negative results.

False-negative cases are characterized by a discrepancy between negative results obtained from CAD and positive results from GeneXpert testing.

### 2.4 Instrument

Xvision, a domestically developed software, uses AI algorithms to analyze data from digital X-rays. Registered by Romania, Slovakia, and Poland, it has ISO 13485 Certification and can generate interpretations for over 17 pathologies using CXR and CT scans. The software has been registered by the Ministries of Health in Romania, Slovakia, and Poland.

TB screening in Romania has adopted the CAD technology, to identify TB from CXR images. The software uses deep learning, a deep neural network, to identify unique characteristics of TB, producing scores ranging from 0 to 100. This technology has proven effective in medical image analysis and other applications.

The GeneXpert instruments used were the Xpert® MTB/RIF Ultra models, known for their versatility and advanced capabilities. These cartridge-based devices have four modules and utilize six colors, enabling efficient detection of MTB and Rifampin resistance mutations within 80 min.

The decision to use GeneXpert as the gold standard for TB diagnosis in this study was based on its well-established accuracy, rapid turnaround time, and its ability to simultaneously detect *Mycobacterium tuberculosis* and rifampin resistance. While we acknowledge its limitations, such as reduced sensitivity in immunocompromised and paucibacillary cases, GeneXpert remains one of the most widely recommended diagnostic tools for active TB by the WHO. Its high specificity and integration into global TB programs made it the most practical and reliable choice for our study setting (3). Furthermore, the AI algorithm threshold of 0.40 was selected based on a combination of prior research findings and internal pilot testing conducted during the initial phases of this project. This value represented an optimal balance between sensitivity and specificity, minimizing false negatives while controlling the rate of false positives. Sensitivity analyses were also performed to validate this threshold against the study population, ensuring its applicability and reliability in vulnerable groups.

### 2.5 Statistical analysis

The data analysis was conducted using MedCalc, a statistical software package specifically designed for biomedical research (Version 22.016) (16), as well as IBM SPSS (Version 26) (17).

The evaluation of sensitivity and specificity is fundamental in assessing the accuracy of test results, but it has various drawbacks when analyzing the accuracy of multiple competing tests (18, 19).

The ROC (Receiver Operating Characteristic) curve was constructed in our study by comparing the AI’s scoring results with the confirmed diagnosis of TB obtained through GeneXpert testing. It is crucial to comprehend the concepts of true positive, true negative, false positive, and false negative, as they arise from the analysis. The ROC curve displays the True Positive Rate (TPR) and False Positive Rate (FPR) at different categorization threshold levels. The metrics can be derived and analyzed from a confusion matrix, which is a method utilized to consolidate the acquired outcomes by comparing accurate and inaccurate predictions in identifying tuberculosis. We employed ROC curve and confusion matrix analysis to produce a precise estimation of the likelihood of TB presence or absence by utilizing AI interpretation (20, 21).

**Figure 1:**
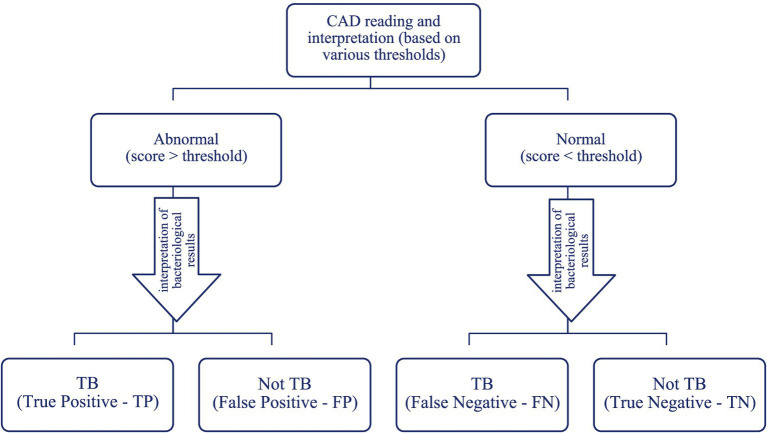
Potential outcomes of computer-assisted detection.

In order to assess the effectiveness of a medical test in correctly identifying individuals who are unwell and accurately rejecting those who are healthy, we utilized two crucial metrics: TPR and FPR (Figure 1).

In this study, sensitivity represents the capacity of a test to accurately detect individuals who have TB, meaning the likelihood that a test result will be positive when the individuals are genuinely sick. It is quantified as the ratio of individuals with a positive outcome among those who are ill, relative to the overall number of individuals who are ill. Increasing the sensitivity of AI-interpreted chest radiography enhances its capacity to accurately detect persons who are unwell, while minimizing the occurrence of false negatives.


$$\mathrm{Sensitivity}=TPR=\frac{TP}{TP+FN}$$

Moreover, specificity is the measure of a test’s accuracy in correctly identifying persons who are healthy and correctly excluding those who do not have TB. It represents the probability that an AI-interpreted CXR will have a low score when the patients are genuinely healthy. The term refers to the ratio of healthy individuals with a negative or poor outcome (such as developing a disease or facing an adverse health event) compared to the overall number of healthy individuals. Greater specificity of a test corresponds to a higher capacity to accurately identify healthy persons, resulting in fewer false positive results.


$$\mathrm{Specificity}=TNR=\frac{TN}{FP+TN}$$

The statistical analysis algorithm adhered to the methodology outlined in Figure 1, considering the scores acquired from the radiological interpretation in conjunction with the results of the GeneXpert testing for both the study group and the control group. The test’s discriminating capacity is considered stronger when there are more realistic findings and fewer errors (20).

The global assessment of CAD reading and interpretation was conducted by determining the area under the ROC curve, which depicts the relationship between sensitivity and (1-specificity). A test is considered more effective as the area under the ROC curve approaches 1, indicating high sensitivity and specificity. Decreasing the range significantly compromises either sensitivity or specificity, resulting in an improvement in one measure at the expense of the other. Thus, when the ROC curve is positioned closer to the upper left corner, it indicates a higher level of overall accuracy for the test (22).

In the evaluation of AI performance for TB diagnosis, the ROC curve analysis includes the assessment of the Youden index (23), also known as the J statistic or J Index. This index plays a crucial role in evaluating the diagnostic test’s ability to correctly identify patients with the condition. It is calculated by subtracting the complement of specificity from sensitivity at a specific threshold of the diagnostic test.


$$J=\max\left\{\mathrm{sensitivit}y_{c}+\mathrm{specificit}y_{c}-1\right\}\;\mathrm{or}\;J=\frac{TP}{TP+FN}+\frac{TN}{TN+FP}-1$$

The value of the Youden index is a useful measure for evaluating the overall performance of a diagnostic test, but it can be influenced by the choice of the cutoff threshold and should be interpreted in conjunction with other performance measures of the test, such as the ROC curve and the values of sensitivity and specificity, in order to obtain a comprehensive assessment of the test’s effectiveness in diagnosis. The value of the criterion corresponding to the Youden Index (J) is the optimal criterion value only when the disease prevalence is 50%, equal weight is given to sensitivity and specificity, and the costs of different decisions are ignored. The J index represents the maximum vertical distance between the ROC curve and the diagonal line (11).

## 3 Results

The main evaluation is conducted on a sample of 404 participants in the study, out of which 202 individuals (50%) have been diagnosed with active TB, identified by active detection during the Romanian screening program. According to Table 1 the participants are the distribution among vulnerable categories is as follows: 358 (88.6%) RR, 14 (3.5%) - PWID, 26 (6.4%) HP, and 6 (1.5%) - PDL.

**Table 1:** Distribution of screening participants by vulnerable groups and TB status.

Vulnerable Group (VG)	Control group		Study group	
	Count	Column *N* %	Count	Column *N* %
HP	13	6.4%	13	6.4%
PDL	3	1.5%	3	1.5%
PWID	7	3.5%	7	3.5%
RR	179	88.6%	179	88.6%
Total	202	100.0%	202	100.0%

The distribution of participants by gender (Supplementary Table A1), for the entire sample (*n* = 404 individuals), reveals a greater percentage of males (58.9%, 238 individuals). However, the composition of each group differs: the study group has a higher proportion of men (74.8%, 151 individuals), while the control group has a higher proportion of women (56.9%, 115 individuals).

Within the entire sample, 91.8% of individuals diagnosed with active TB were aged 35 or older. Specifically, about half of the 404 participants were within the age range of 45 to 64 (Supplementary Table A2).

According to the data 83.2% (336 individuals) of the participants are currently not participating in the labor market, where as 15.8% (64 individuals) are actively engaged in it. Additionally, 1% (4 individuals) are currently unemployed (Supplementary Table A3).

Regarding the CAD reading and interpretation results, the study group has an average TB score of 0.77 and a median value of 0.92. The control group, on the other hand, has an average value of 0.13, with a median of 0.059. Simultaneously, anomalous values are detected within both groups.

**Figure 2:**
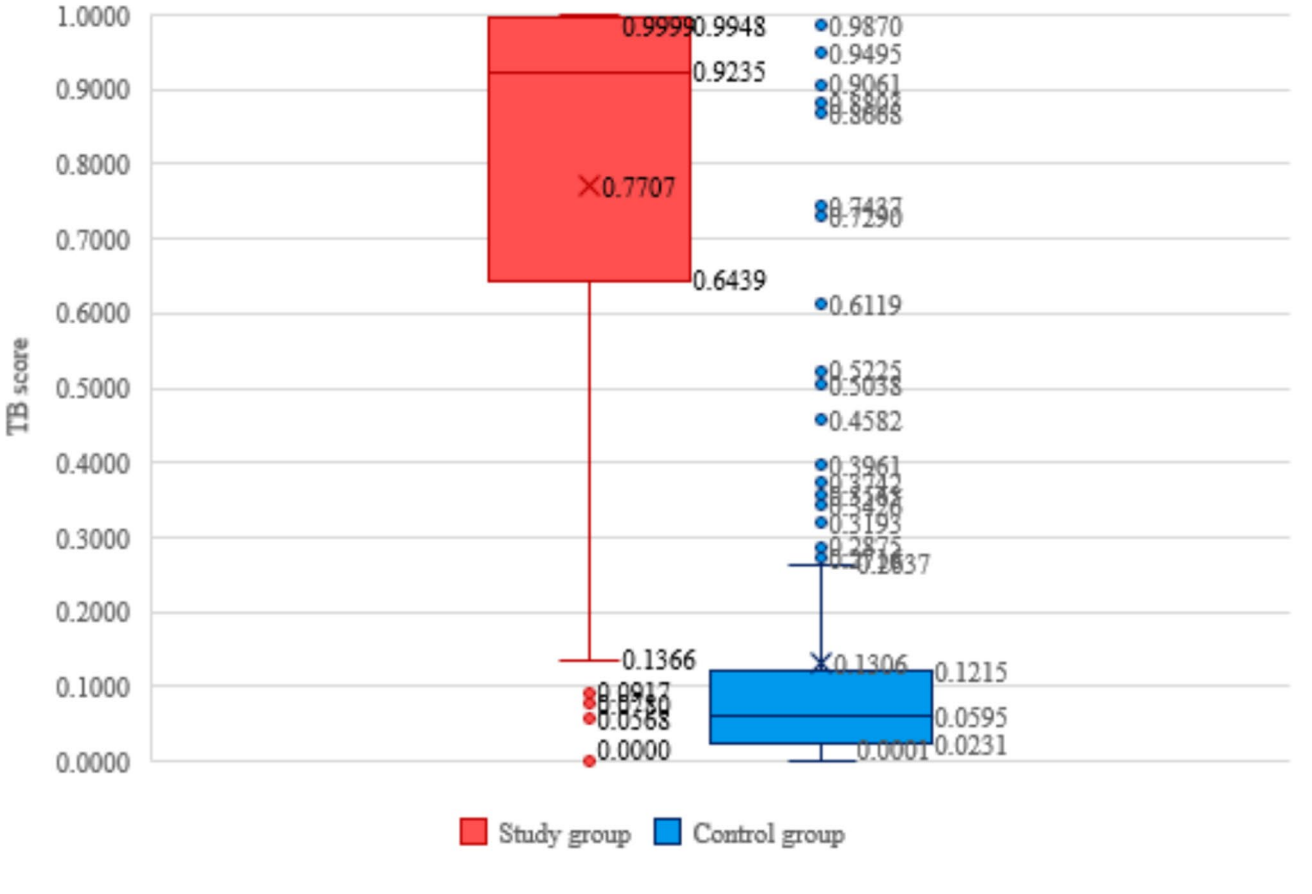
Distribution of participants vulnerable group categories, TB score and TB status.

Hence, considering the study’s context and particularities, we will examine whether the outlier values seen in both the study group (positive) and the control group (negative) have any clinical significance (Figure 2).

Both groups exhibit instances of asymmetry (Figure 2). Among the members of the study group, there is a distribution of TB scores that exhibits a negative asymmetry. Specifically, 13 out of the 202 patients with active TB had a TB score below 0.1366, which is considered an outlier value. Regarding the control group, it is seen that there is a positively skewed distribution, where TB scores exceeding 0.2637 are considered outlier values.

In our study, the comparison of CAD operation in the two groups with the results of GeneXpert tests and subsequent medical evaluations (see Table 2) reveals that 42.8% of the outcomes are true positives and 46.2% are true negatives. This implies that 89% of the screened individuals benefitted from accurate assessments utilizing AI alongside radiological examinations. The double number of false negative results brought by chest radiography with AI, 7.2% compared to 3.7%, is a percentage that we believe has to be analyzed in accordance with the limitations of chest radiological examination in terms of sensitivity and specificity.

**Table 2:** Distribution of participants in the screening according to the CAD result.

	Control group		Study group		Total	
	Count	Column *N* %	Count	Column *N* %	Count	Column *N* %
FN	0	0.0%	29	14.4%	29	7.2%
FP	15	7.4%	0	0.0%	15	3.7%
TN	187	92.6%	0	0.0%	187	46.3%
TP	0	0.0%	173	85.6%	173	42.8%
Total	202	100.0%	202	100.0%	404	100.0%

**Figure 3:**
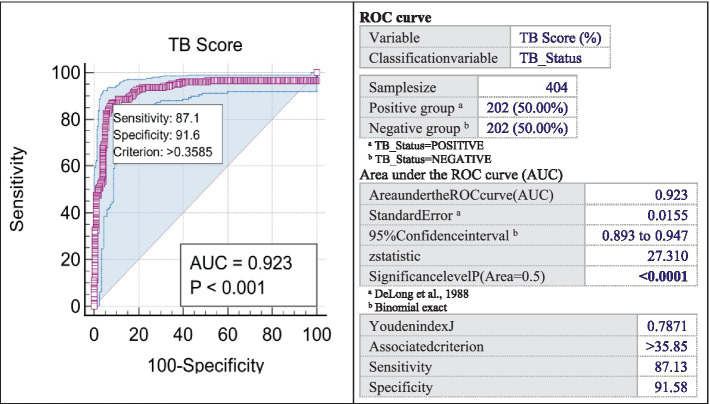
ROC curve.

The next stage of analysis consisted in evaluating the sensitivity and specificity by utilizing the ROC curve. The AUC (Area Under the Curve) quantifies the overall effectiveness of AI in differentiating between positive and negative instances of TB. A test’s diagnostic performance improves as the AUC increases (22). The findings from our research, which involved 404 participants, reveal that the AUC value of 0.923 in the ROC curve used for AI assessment in diagnosing TB indicates excellent performance in accurately differentiating between patients who are positive and negative for this condition. A test with an AUC close to 1 indicates a strong discriminative ability and superior diagnostic performance. Therefore, the AUC value of 0.923 (95% CI: 0.893–0.947; significance level *p* < 0.0001) demonstrates that the test has a high capacity to accurately detect individuals with tuberculosis and, at the same time, to exclude those without the disease, with a sensitivity of 87.1% and a specificity of 91.6% (Figure 3).

The Youden index *J* = 0.781, obtained in our study, signifies a substantial test performance in effectively discriminating between positive and negative outcomes (23). This indicates a proficient ability in CAD to differentiate between positive and negative instances of TB. The criterion, with a value greater than 0.3585, serves as an accurate threshold for classifying the test results (24).

The CAD sensitivity reached in our study is 87.13%, demonstrating the AI’s capacity to accurately distinguish true positive cases from the overall positive cases within the group of individuals suspected of TB. Furthermore, the software demonstrates a specificity of 91.58%. This indicates the test’s capacity to accurately identify the true negative cases among all individuals present during the screening.

**Figure 4:**
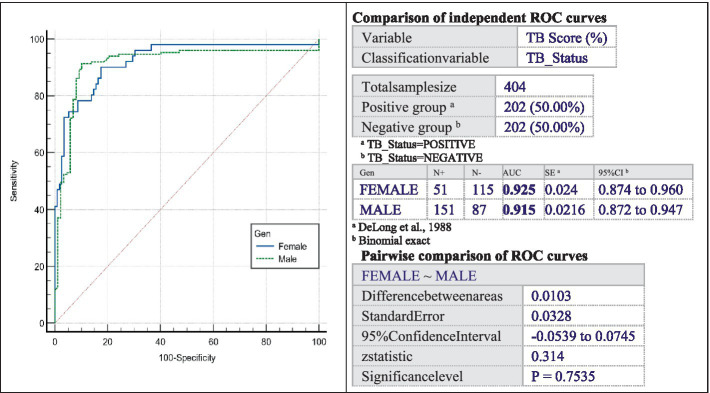
Curves of male and female.

These findings indicate that the application of CAD for the imaging diagnosis of TB has an adequate level of performance in detecting the disease, showing a remarkable ability to accurately identify both positive and negative cases, with no significant difference between women and men as shown in Figure 4 (p=0.7535>0.05).

## 4 Discussions

### 4.1 Analysis of threshold value

Early detection of TB, particularly in vulnerable groups in rural areas, is crucial. This is because outbreaks in these communities can lead to a higher risk of disease transmission through migration. In particular, 9.3% of TB cases in European states are found in migrants from Romania (25).

The use of AI software in radiological assessment is being defined as active detection and is becoming an important part of TB control efforts in several nations worldwide. Our research highlights the importance of using AI software in diagnosing TB alongside bacteriological diagnosis. This study focuses on the new program X-vision, which is now available on the international market for such research. The intervention’s uniqueness stems from its broad breadth and the specific characteristics of the target group. The AI program is employed to assess over 90,000 individuals, a feature unparalleled in existing literature.

When discussing the four vulnerable groups selected in our study, the homeless individuals, the group is characterized by a lack of stable income, absence of health insurance, and occasionally a lack of social help. Individuals who take injectable medicines are at a higher risk of acquiring airborne infections due to the prevalence of Hepatitis and Human immunodeficiency viruses among this population. Rural residents in many parts of Romania experience inferior socio-economic status, restricted access to healthcare facilities, inadequate health knowledge, and insufficient information for effective preventative actions, leading to a higher prevalence of infectious diseases. Finally, individuals who are incarcerated reside in cramped conditions and are exposed to significant pressures.

The selected threshold of 0.40, was chosen to minimize the delay between the time of evaluation and the reading of CXR by the doctors. The objective of our project was to reduce the occurrence of false negative instances generated by AI in CXR, by prioritizing the utilization of supplementary diagnostic approaches. The selection of the threshold score entails an inherent trade-off; opting for a lower threshold score will increase the tool’s sensitivity in identifying actual TB cases within the screened population, but it will also lead to increased costs due to unnecessary follow-up diagnostic tests resulting from reduced specificity.

Conversely, opting for a higher threshold score is expected to enhance the identification of more severe cases and reduce the need for extensive and expensive follow-up diagnostic testing. However, this approach’s reduced sensitivity may result in instances being overlooked or undetected.

A study conducted in South Africa evaluated the efficacy of CAD in diagnosing TB through digital imaging methods. The research employed a threshold exceeding 60, with confirmation through GeneXpert testing and liquid cultures. CXR screening identified a comparable number of previously undiagnosed TB cases relative to symptom-based screening, while requiring fewer diagnostic tests. The objective aspect of CXR screening can enhance case detection in clinics (26).

The analysis of patients in our study revealed that using the software between the thresholds of 0.65 and 0.4 only detected one positive case. This finding has no impact on identifying true positive instances but enhances the accuracy in detecting negative cases.

When considering gender analysis, there are no significant differences found in the comparison analysis of the ROC curve based on sex (significance level, *p* = 0.7535 > 0.05). With an AUCF of 0.925 and an AUCM of 0.915, the test demonstrates great accuracy in properly identifying individuals with TB and excluding those without the disease, regardless of their gender.

The choice of the threshold is heavily influenced by the specific algorithm and the particular context in which it is applied. For instance, the threshold needed to achieve at least 90% sensitivity varies for different software: InferRead DR requires a threshold below 0.34, CAD4TB below 0.50, qXR and Lunit INSIGHT CXR below 0.60, and JF CX R-1 below 0.93. According to the study, all five AI algorithms demonstrated a sensitivity of over 90% when automatically reading chest X-rays. This promising result has the potential to significantly reduce the number of additional diagnostic tests required. However, it is important to carefully consider the choice of the threshold. A sensitivity of 70% may restrict the use of diagnostic tests, potentially leading to the omission of up to 30% of TB cases (25).

A study conducted in Vietnam involved the simultaneous interpretation of all CXR in parallel with CAD software called qXR 3.0. The program was set at a threshold score of ≥0.60 which exhibited a high level of sensitivity and a low level of specificity in detecting TB cases verified by GeneXpert. The concordance between the physician and the results of the CAD from the CXR improved when the software was trained (27).

### 4.2 Implementation challenges and broader implications

While AI-based diagnostic tools like X-Vision show promising results in tuberculosis screening, their implementation in vulnerable populations faces several challenges. Limited access to healthcare facilities, insufficient infrastructure, and digital illiteracy may hinder the adoption of AI technologies, particularly in remote rural areas and marginalized groups. Additionally, discrepancies in AI performance may arise due to differences in data quality, variations in imaging equipment, or incomplete training datasets, which could affect diagnostic accuracy. Addressing these barriers requires investments in infrastructure, training programs, and community engagement initiatives to ensure equitable access and effective deployment of AI systems. Furthermore, the findings of this study have broader implications for other high-burden TB countries with similar socio-economic challenges. The integration of AI with rapid molecular tests like GeneXpert could serve as a scalable and cost-effective model for TB control programs globally, particularly in regions where access to specialist radiologists and advanced diagnostic tools is limited. AI in TB diagnosis currently allows for the use of radiologist expertise only in borderline instances, both medically and imaging-wise, and is a solution that medical systems must develop toward.

### 4.3 ROC curve analysis

The ROC curve in this study shows a sensitivity of 87.1% and a specificity of 91.6%, with an AUCF of 0.925 and an AUCM of 0.915. The test exhibits high accuracy in identifying individuals with TB and ruling out those without the disease, irrespective of gender, highlighting the enhanced performance of the Xvision software in detecting tuberculosis cases. Regarding the gender analysis for the ROC curve in this study, it does not highlight significant differences in the comparative analysis by sex (significance level, *p* = 0.7535 > 0.05).

During an analysis comparing the AUC curves of 5 AI algorithms, it was found that all of them performed admirably. Notably, qXR and CAD4TB stood out as the top performers (21). For the majority of threshold anomaly scores (excluding very low or high scores), the sensitivity of JF CXR-1 remained consistently above 90%. Similarly, CAD4TB, Lunit INSIGHT CXr, and qXR maintained a sensitivity of approximately 80% for most thresholds (≤0.8), before experiencing a rapid decline. The study conducted by QIN ZZ and colleagues compared three AI software programs, namely Lunit, qXR, and CAD4TB (28). The study found that while only qXR and CAD4TB met the technical requirements of the FIND TPP for a triage test with a sensitivity of ≥95% and specificity of ≥80%, all three products demonstrated similar performance. Consequently, all three software programs can be employed to reduce the need for additional diagnostic tests while maintaining high sensitivity, ultimately leading to cost savings. Software such as CAD4Tb and qXR, particularly in their updated versions, exhibit clear superiority and consistently outperform human interpretation in terms of diagnostic accuracy.

Another study compared the results of scoring against radiologist readings and WHO’s Target Product Profile (TPP) for a TB triage test, for 12,890 chest X-rays. Both newer versions significantly outperformed their predecessors in terms of AUC: CAD4TB version 6 (0.823 [0.816–0.830]), version 7 (0.903 [0.897–0.908]) and qXR version 2 (0.872 [0.866–0.878]), version 3 (0.906 [0.901–0.911]). All products equaled or surpassed the human radiologist performance with improvements in triage ability in newer versions. In our study AUC was 0.925, higher than the values obtained in the study conducted by Zhi Zhen Qin (28).

### 4.4 Analysis of false negative and false positive outcomes

Aberrant notions in the analysis of false positive/false negative data in clinical trials share commonalities in their impact on result interpretation, but are distinct concepts encountered in different situations and fields. An in-depth examination of these values is necessary to avoid inaccurate conclusions or choices in this study.

The confusion matrix indicates that our study achieved an accuracy of 89.1% (TN + TP), a specificity of 91.6%, reflecting improved precision in TB detection, and a sensitivity of 87.1%. The test has an error rate of 10.9% (FN + FP) and a negative predictive value of 92.6% (TN), showing its ability in accurately ruling out negative circumstances. The specific interpretation depends on the severity of the consequences for incorrect negative and positive outcomes within the particular context of the clinical experiment. The outlier values were assessed in comparison to the findings of the bacteriological examination.

Out of the 202 patients in the study group that tested positive for GeneXpert during screening, 29 patients (14.4% of the study group and 7.2% of the total) had false negative findings for TB scoring, with 13 of them having TB score values below 0.10. Out of those 29 FN patients, 9 had a previous TB disease and two out had no history of TB and were not later confirmed to have TB during medical and bacteriological examinations. As a result, 27 patients with FN TB scores but positive sputum at GeneXpert were reevaluated.

In the control group, 15 patients had false positive TB scores (> 0.40) but tested negative for TB on the GeneXpert test. These false positive cases accounted for 7.4% of the control group and 3.7% of the total 404 persons analyzed. We note that the sample was randomly selected from all participants registered in the screening and had negative TB sputum. The results of the descriptive analysis reveal notable aspects of the dataset, with a particular focus on extremely high values. In the case of the control group (202 participants with negative TB score), while the mean (0.1306) provides insight into the central tendency, the Kurtosis (7.8877) and skewness (2.8271) values indicate a pronounced peak in the distribution and a strong positive skewness, suggesting the presence of extreme values. In addition, outlier values are underscored by a substantial standard deviation (0.2052). The minimum of 0.0001 and the maximum of 0.9987 illustrate significant variability within the dataset, and the high Kurtosis coefficient implies that the distribution may contain extreme values that are either positively or negatively skewed. These findings underscore the distinctive nature of the dataset, which is characterized by a pronounced concentration around the mean and the presence of significant outlier values.

The 15 scoring values exceeding 0.40 were thoroughly examined as they were within the false positive range. Out of the 15 patients, 3 had a previous history of TB with scores of 0.9987, 0.9495, and 0.8668. These elevated values may indicate the presence of pulmonary sequel lesions with recent inflammatory features due to exacerbations.

Not surprisingly, we also found higher CAD scores in individuals experiencing TB recurrence, in such circumstances, it may be beneficial to consider including them in the criteria for predicting recurrence.

The low CAD score of the 7 individuals with a TB history, although testing positive for TB in sputum using GeneXpert, does not indicate active TB, results also supported by the study conducted by Grant Theron on 238 patients who tested positive on GeneXpert and treated for TB and retested positive on GeneXpert (29). The high incidence of TB in 12 patients without a prior TB history highlights the need to update the AI scoring algorithm in X-Vision to better differentiate between radiological images of concurrent infections, which occur alongside other conditions, and those of active TB. The limitation of the evaluation method, which combines imaging analysis and bacteriological testing via GeneXpert, has an error rate of less than 1%, a statistically acceptable threshold. Upon analyzing the entire batch, it is evident that 7.2% of individuals exhibit FN results, whilst 3.7% display FP results. The study’s main goal was to detect TB, with a focus on the costs of testing. These costs are high initially but decrease dramatically in the long term when compared to the expenses incurred due to false negative instances.

Compared data with Melendez’s study, which assessed the initial version of CAD for TB in over 38,000 individuals, the ROC curve analysis showed a software specificity of 55.71% (95% CI 55.21–56.20) and a negative predictive value of 99.98% (95% CI 99.95–99.99) at a sensitivity of 95%. The ROC curve’s area was 0.90 with a 95% confidence interval of 0.86–0.93 (30). In our study ROC curve analysis indicate an AUC of 0.923 (95% CI:0.893–0.947; significance level *p* < 0.0001) with sensitivity 87.1, specificity 91.6 and criterion >0.3585.

### 4.5 Study limitation

Our study is limited by the software’s inability to differentiate lung lesions present in new TB cases from specific TB lung lesions in relapsed cases. Training the software for a new working variant would allow for the reduction of false negative cases, which pose the greatest challenges in terms of TB transmission perspective. Another limitation of the statistical analysis arises from the small sample sizes of drug users and homeless patients, which prevented distinct identification in the diagnostic algorithms for these categories.

## 5 Conclusion

The combination of CXR with AI scoring and GeneXpert testing is very advantageous for the screening of a population exposed to risk, when we talk about the active detection of TB. The primary objective of this technology is not merely to reduce costs, but to provide significant assistance in the detection of cases and the management of infections. Additionally, it streamlines the triage process by lowering the costs of triage algorithms, reducing human error, and enhancing the role of radiological examinations in identifying lung lesions. Given that the sensitivity and specificity of CXR are known to slightly exceed the 50% threshold, this technology offers valuable support in improving diagnostic accuracy.

A tool like this could prove particularly valuable in situations where there is a significant constraint on human resources, especially doctors, and in areas where access to medical services is limited. The test’s AUC is 0.924 with a 95% confidence interval (CI) of 0.913–0.964, indicating a remarkable ability to accurately detect individuals with TB and also correctly exclude those which are not affected.

## Data Availability

The raw data supporting the conclusions of this article will be made available by the authors, without undue reservation.
