# Supplementary material for: Artificial intelligence: a useful tool in active tuberculosis screening among vulnerable groups in Romania - advantages and limitations
Source: Front Public Health. 2025 Feb 7;13:1433450. doi: 10.3389/fpubh.2025.1433450 (PMC11842365; doi:10.3389/fpubh.2025.1433450)
Supplement: Supplementary file 1 [file Table_1.DOCX]

Supplementary Material

Artificial intelligence: a useful tool in active tuberculosis screening among vulnerable groups in Romania - advantages and limitations

Beatrice Mahler ^1,2,4^, Alexandru Stoichiță ^1,2,4*^, Dragoș Băiceanu ^3,4^, Traian-Constantin Panciu^3,4^, Dragoș Dendrino^4,9^, Mihaela Mihai^4,5*^, Raluca Bobocea^2^, Elmira Ibraim ^3,4^, Mara Bălteanu^2,6^, Oana Popescu^2,4^, Mădălina Oana Burecu^2,4^, Ioana Mădălina Moșteanu ^4,7^, Vanessa Veronese^8^, Radu Matache^1,2^, Ioana Munteanu^2,4,6^, Cristina Popa^2,4^, Antonela Dragomir^1,2,4^

*** Correspondence:**Mihaela Mihai: [mihai.mihaela@csie.ase.ro](mailto:mihai.mihaela@csie.ase.ro);
Alexandru Stoichiță: [alexandru.stoichita@drd.umfcd.ro](mailto:alexandru.stoichita@drd.umfcd.ro)

# Supplementary Tables

Table A.1 Distribution of screening participants by gender and TB status

| Sex | **Control group** | | **Study group** | | **Total** | |
| --- | --- | --- | --- | --- | --- | --- |
|  | Count | Column N % | Count | **Column N %** | Count | Column N % |
| FEMALE | 115 | 56.9% | 51 | 25.2% | 166 | 41.1% |
| MALE | 87 | 43.1% | 151 | 74.8% | 238 | 58.9% |
| Total | 202 | 100.0% | 202 | 100.0% | 404 | 100.0% |

Table A.2 Distribution of screening participants by sex and age group

| **Sex** | **Age Category**  **(years)** | **Control group** | | **Study group** | | **Total** | |
| --- | --- | --- | --- | --- | --- | --- | --- |
|  |  | **Count** | **Column N %** | **Count** | **Column N %** | **Count** | **Column N %** |
| FEMALE | 20–24 | 2 | 1.7% | 2 | 3.9% | 4 | 2.4% |
|  | 25–34 | 14 | 12.2% | 4 | 7.8% | 18 | 10.8% |
|  | 35–44 | 13 | 11.3% | 7 | 13.7% | 20 | 12.0% |
|  | 45–54 | 22 | 19.1% | 10 | 19.6% | 32 | 19.3% |
|  | 55–64 | 29 | 25.2% | 7 | 13.7% | 36 | 21.7% |
|  | 65–74 | 21 | 18.3% | 11 | 21.6% | 32 | 19.3% |
|  | 75–84 | 13 | 11.3% | 9 | 17.6% | 22 | 13.3% |
|  | >=85 | 1 | 0.9% | 1 | 2.0% | 2 | 1.2% |
|  | Total | 115 | 100.0% | 51 | 100.0% | 166 | 100.0% |
| MALE | 20–24 | 2 | 2.3% | 0 | 0.0% | 2 | 0.8% |
|  | 25–34 | 4 | 4.6% | 9 | 6.0% | 13 | 5.5% |
|  | 35–44 | 21 | 24.1% | 23 | 15.2% | 44 | 18.5% |
|  | 45–54 | 17 | 19.5% | 45 | 29.8% | 62 | 26.1% |
|  | 55–64 | 17 | 19.5% | 37 | 24.5% | 54 | 22.7% |
|  | 65–74 | 21 | 24.1% | 23 | 15.2% | 44 | 18.5% |
|  | 75–84 | 3 | 3.4% | 12 | 7.9% | 15 | 6.3% |
|  | >=85 | 2 | 2.3% | 2 | 1.3% | 4 | 1.7% |
|  | Total | 87 | 100.0% | 151 | 100.0% | 238 | 100.0% |
| Total | 20–24 | 4 | 2.0% | 2 | 1.0% | 6 | 1.5% |
|  | 25–34 | 18 | 8.9% | 13 | 6.4% | 31 | 7.7% |
|  | 35–44 | 34 | 16.8% | 30 | 14.9% | 64 | 15.8% |
|  | 45–54 | 39 | 19.3% | 55 | 27.2% | 94 | 23.3% |
|  | 55–64 | 46 | 22.8% | 44 | 21.8% | 90 | 22.3% |
|  | 65–74 | 42 | 20.8% | 34 | 16.8% | 76 | 18.8% |
|  | 75–84 | 16 | 7.9% | 21 | 10.4% | 37 | 9.2% |
|  | >=85 | 3 | 1.5% | 3 | 1.5% | 6 | 1.5% |
|  | Total | 202 | 100.0% | 202 | 100.0% | 404 | 100.0% |

Table A.3 Distribution of screening participants by, sex, labor market status and TB status

| **Sex** | **Age Category**  **(years)** | **Control group** | | **Study group** | | **Total** | |
| --- | --- | --- | --- | --- | --- | --- | --- |
|  |  | **Count** | **Column N %** | **Count** | **Column N %** | **Count** | **Column N %** |
| FEMALE | EMPLOYEE | 20 | 17.4% | 5 | 9.8% | 25 | 15.1% |
|  | INACTIVE | 94 | 81.7% | 45 | 88.2% | 139 | 83.7% |
|  | UNEMPLOYED | 1 | 0.9% | 1 | 2.0% | 2 | 1.2% |
|  | Total | 115 | 100.0% | 51 | 100.0% | 166 | 100.0% |
| MALE | EMPLOYEE | 17 | 19.5% | 22 | 14.6% | 39 | 16.4% |
|  | INACTIVE | 70 | 80.5% | 127 | 84.1% | 197 | 82.8% |
|  | UNEMPLOYED | 0 | 0.0% | 2 | 1.3% | 2 | 0.8% |
|  | Total | 87 | 100.0% | 151 | 100.0% | 238 | 100.0% |
| Total | EMPLOYEE | 37 | 18.3% | 27 | 13.4% | 64 | 15.8% |
|  | INACTIVE | 164 | 81.2% | 172 | 85.1% | 336 | 83.2% |
|  | UNEMPLOYED | 1 | 0.5% | 3 | 1.5% | 4 | 1.0% |
|  | Total | 202 | 100.0% | 202 | 100.0% | 404 | 100.0% |
